# Color, Structure, and Thermal Stability of Alginate Films with Raspberry and/or Black Currant Seed Oils

**DOI:** 10.3390/molecules30020245

**Published:** 2025-01-09

**Authors:** Jolanta Kowalonek, Bogna Łukomska, Aleksandra Szydłowska-Czerniak

**Affiliations:** 1 Department of Biomedical and Polymer Chemistry, Faculty of Chemistry, Nicolaus Copernicus University in Toruń, Gagarina 7, 87-100 Toruń, Poland; bognalukomska@abs.umk.pl; 2 Department of Analytical Chemistry and Applied Spectroscopy, Faculty of Chemistry, Nicolaus Copernicus University in Toruń, Gagarina 7, 87-100 Toruń, Poland

**Keywords:** bioactive-rich oils, alginate film, optical properties, structure, thermal stability

## Abstract

In this study, biodegradable and active films based on sodium alginate incorporated with different concentrations of oils (25% and 50%) from fruit seeds were developed for potential applications in food packaging. The ultraviolet and visible (UV-VIS) spectra of raspberry seed oil (RSO) and black currant seed oil (BCSO) indicated differences in bioactive compounds, such as tocopherols, phenolic compounds, carotenoids, chlorophyll, and oxidative status (amounts of dienes, trienes, and tetraenes) of active components added to alginate films. The study encompassed the color, structure, and thermal stability analysis of sodium alginate films incorporated with RSO and BCSO and their mixtures. The color of alginate films before and after the addition of oils from both fruit seeds was evaluated by measuring color coordinates in the CIELab color space: L* (lightness), a* (red-green), and b* (yellow-blue). The lightness values ranged between 94.21 and 95.08, and the redness values varied from −2.20 to −2.65, slightly decreasing for the films enriched with oils. In contrast, yellowness values ranged between 2.93 and 5.80 for the obtained active materials, significantly increasing compared to the control alginate film (L* = 95.48, a* = −1.92, and b* = −0.14). Changes in the structure and morphology of the alginate films after incorporating bioactive-rich oils were observed using scanning electron microscopy (SEM). Films with RSO and oil mixtures had more developed surfaces than films with BCSO. Moreover, the cross-sections of the films with RSO showed holes evenly distributed inside the films, indicating traces of volatile compounds. Thermal decomposition of the alginate films loaded with oils showed five separate stages (to 125 °C, 125–300 °C, 310–410 °C, 410–510 °C, and 750–1000 °C, respectively) related to the oil and surfactant decomposition. The shape of the thermogravimetric curves did not depend on the oil type. The added oils reduced the efficiency of alginate decomposition in the first stage. The obtained results showed that new functional and thermally stable food packaging films based on sodium alginate with a visual appearance acceptable to consumers could be produced by utilizing oils from fruit seed residues.

## 1. Introduction

Plastics and other polymers, glass, and paper are among the most commonly used traditional packaging materials to protect food and cosmetic products and ensure their freshness and quality. In particular, plastic plays a unique role in food packaging and cosmetic products due to its lighter weight, affordability, design flexibility, chemical resistance, ability to seal in heat, and ability to protect finished food and cosmetic products during the supply chain process. However, the growing consumer preference for natural food and cosmetic products, including regarding preservation at the packaging stage, as well as some major disadvantages of plastic, such as its non-biodegradable and non-renewable nature, has driven innovation in alternative protective methods that use biopolymers sourced from sustainable materials or industrial byproducts. Various biopolymers, including polysaccharides (cellulose, chitin and chitosan, pectin, starch, alginate, xanthan gum), proteins (collagen, gelatin, soy protein), and lipids have recently been applied to the production of films and coatings due to their functional packaging properties [1]. Moreover, because they are environmentally friendly and economical sources of biopolymers, agro waste or byproducts can be used to prepare films and coatings with promising film-forming properties. In recent years, packaging systems comprised of natural polymers have been loaded with essential oils [2,3,4,5,6] and antioxidant extracts from food wastes [7,8,9,10,11], which enhances their antioxidant and antibacterial properties. These types of films with antioxidant and antibacterial properties, called active packaging, should contain biopolymers and natural active additives. Active ingredients incorporated into edible films based on safe and biodegradable biopolymers improve their physicochemical and functional properties, including natural antioxidants, pigments, flavors, vitamins, nutraceuticals, and polypeptides. Therefore, active packaging goes beyond the traditional role of packaging by imparting specific, intentional functionality to the packaging system. Nowadays, active edible films are designed to extend the shelf life of packaged products, impart post-package processing, and improve food and cosmetics safety and quality.

On the other hand, oils recovered from various seeds, including strawberry, blackberry, black currant, raspberry, cranberry, grape, and apple—which are byproducts of fruit processing—contain polyphenolic compounds (phenolic acids, flavonoids, and anthocyanins), tocopherols, carotenoids, vitamins, and phytosterols with favorable antioxidant, antimicrobial, and anti-inflammatory properties.

In recent years, various oils extracted from seed wastes have been incorporated into edible films to improve their functional properties. Grape seed oil added to hydroxypropyl methylcellulose films and starch/konjac gum composite films in both its pure form and as nanoliposomes improved their mechanical, color, water vapor barrier, and antioxidant and antibacterial properties [12,13]. Moreover, Marudova et al. [14] reported that rosehip seed oil incorporated into chitosan films at different concentrations ranging between 1% and 5% affected the physicochemical, antioxidant, and antifungal properties. However, thermal analysis confirmed that the presence of oil did not influence the film stability at temperatures up to 100 °C. Chitosan-based edible films loaded with *Berberis crataegina* DC.’s seed oil and fruit extract were also fabricated to increase antioxidant, antimicrobial, and anti-quorum sensing activities [15]. However, fortifying soy protein isolate edible films into omega-rich purslane seed oil at different concentrations of 1%, 2%, and 3% improved functional, mechanical, thermostability, antioxidant, antibacterial, and color qualities [16]. Additionally, borage seed oil rich in unsaturated fatty acids and phenolic compounds, including rosmarinic acid, p-coumaric acids, and tocopherols, was used as a chemical modifier to improve the functional properties of films prepared from the barnyard millet starch [16]. The results obtained by the previously mentioned authors suggest that oils recovered from various seeds—which are high in nutrients, including unsaturated fatty acids, natural antioxidants, pigments, phytosterols, minerals, aromatic substances, bacterial and viral inhibitory substances—can be crucial candidates for use as active agents in food packaging based on natural polymers. Among them, oils obtained from black currant and raspberry seeds, which are the primary wastes when obtaining juices from these fruits, can attract extensive attention as active ingredients in biodegradable active films. These oils contain high amounts of unsaturated fatty acids (about 94% and 89% for raspberry and black currant oils, respectively) with desirable omega 6 to omega 3 ratios (1.5 and 3.3, respectively) [17]. Furthermore, they contain similar amounts of other bioactive components, such as polyphenols, tocopherols, carotenoids, and phytosterols [17,18,19,20,21,22].

It is worth noting that sodium alginate, generally recognized as a safe polysaccharide originating from brown algae, is often applied in edible films and coatings due to its good film-forming properties, excellent biocompatibility, degradability, and nontoxicity [23]. In most current research, various additives, such as *Lepidium sativum* (Garden cress) extract [24], purple onion peel extract, butterfly-pea flower extract [25], grape seed phenolics recovered from winemaking byproducts [26], and ylang-ylang essential oil [4], have been used to improve alginate films’ active properties, including its antioxidant potential and antimicrobial activity. However, alginates interact with phenol compounds mainly by forming hydrogen bonds involving the hydroxyl groups of phenols and hydroxyl and carboxyl groups of alginates. For instance, proanthocyanidin-rich grape seed phenolics encapsulated in alginate-Ca^++^ or alginate-Ca^++^-chitosan decreased bioactivity by about 60% due to interactions between phenolics and the carrier polymers [26]. In contrast, Santos and Martins [25] observed that a mixture of the purple onion peel and butterfly-pea flower extracts enabled the development of materials with better packaging performance, as their physical, mechanical, and barrier characteristics were modified due to chemical interactions between phenolic compounds and biopolymers. Moreover, the interaction between these extracts and the alginate positively modified the structure of the films, increasing their melting temperature. For this reason, further research is needed to optimize the formulation and application of alginate-based packaging materials for the preservation of food and cosmetic products by considering the interactions between the active compounds, the product matrix, and the packaging material.

In our previous study, raspberry seed oil (RSO) and black currant seed oil (BCSO) were used as reinforcing agents to enhance the physicochemical characteristics, mechanical strength, and antioxidant and antibacterial barrier properties of sodium alginate films [27]. These alginate films with a higher content (50%) of RSO and BCSO were characterized by a significantly higher antiradical scavenging activity determined by 2,2-diphenyl-1-picrylhydrazyl assay (DPPH = 128.68 and 21.43 μmol Trolox/100 g, respectively) and improved physicochemical properties compared to films with a lower content (25%) of the added oils (64.71 and 6.78 μmol Trolox/100 g, respectively). In particular, films with RSO exhibited excellent antioxidant properties. On the contrary, the obtained alginate films loaded with oils from fruit seeds had no antibacterial activity against *Pseudomonas aeruginosa* and *Staphylococcus aureus*. However, the activity of the investigated films against *Escherichia coli* was very weak, whereas the growth of *Bacillus subtilis* was partially inhibited. Interestingly, there was no significant difference between the actions of the studied oils recovered from byproducts of the fruit industry and the mentioned bacteria. Specifically, the distribution and concentration of inter- and intra-molecular interactions between alginate macromolecules and the components of added fruit seed oils were critical in determining these films’ physicochemical, antioxidant, and antimicrobial performances [27]. Moreover, the enrichment of alginate film with functional ingredients such as oils from fruit wastes (seeds) is appropriate for the management of seed wastes following a valorization-based approach, which states that seed residues are an important potential source of oils with valuable natural components.

However, to the best of our knowledge, the effect of adding different concentrations of these two oils from fruit seeds on the optical, structural, and thermal properties of alginate-based materials has not been reported in the literature in recent years.

Therefore, this study was designed to prepare biodegradable alginate-based films loaded with different concentrations of bioactive-rich raspberry seed oil (RSO), black currant seed oil (BCSO), and their mixtures to evaluate for the first time the impact of these active additives on the optical, morphological structure, and thermal properties of the fabricated films. In addition, the bioactive components, such as tocopherols, phenolic compounds, pigments, and products of oxidative processes present in oils from fruit seeds before addition to alginate films, were monitored using UV-VIS spectra.

This research contributed an innovative approach to developing sustainable and active packaging materials by incorporating hydrophobic antioxidant compounds present in oils recovered from seeds, which are fruit industry wastes, into hydrophilic biopolymer matrices such as sodium alginate.

## 2. Results and Discussion

### 2.1. UV-VIS Absorption Spectra of Raspberry Seed Oil and Black Currant Seed Oil

**Figure 1:**
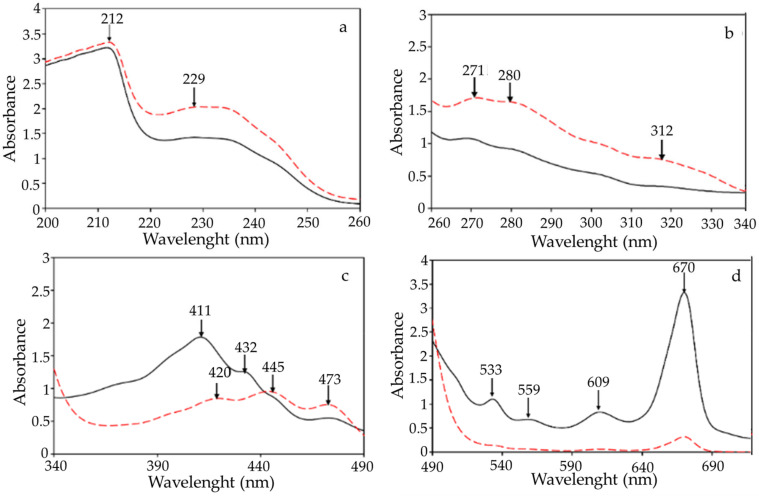
UV-VIS spectra of raspberry seed oil (RSO) (red dashed line) and black currant seed oil (BCSO) (black solid line) diluted in *n*-hexane in different UV-VIS ranges; dilution of oil in *n*-hexane (**a**) 1:500; (**b**) 1:50; (**c**) 1:10; (**d**) 1:1.

The UV-VIS spectra within the 200 to 800 nm range of the RSO and BCSO are presented in Figure 1. These UV-VIS absorption spectra profiles measured for the two studied oils confirm that the content of chemical compounds from specific groups, including antioxidants and oxidative products, varies depending on the oil and is usually specific to a given type of oil. As can be seen, the UV-VIS spectra of both oils recovered from fruit seeds revealed a peak at 212 nm, indicative of π–π * transitions (Figure 1a). Furthermore, the broad bands spanning 220–250 nm were associated with carboxylic and phenolic acid groups. In particular, RSO had higher amounts of oxidative products than BCSO due to more intensive absorbance between 220 and 234 nm, which is characteristic of oxidation compounds. On the other hand, absorbance maxima between 270 and 290 nm (Figure 1b), often considered a purity criterion of oil, suggest that RSO, in comparison with BCSO, contained higher amounts of tocopherols with antioxidant properties [28]. Many authors reported higher concentrations of total tocochromanols in various raspberry seed oils (1980.0–3019.2 mg/kg) than those in oils from black currant seeds cultivated in different regions of Europe and Canada (811.4–2458.0 mg/kg) [17,18,19,20,22]. The major tocopherol in both oils was the γ–homolog, above 50% of the total tocopherols.

Moreover, based on the recorded spectra, various classes of phenolic acids and flavonoids, especially those with conjugated rings, such as flavones and flavonols, absorb in the 300–500 nm (Figure 1b,c), can be found in the compositions of the investigated oils. However, more intense absorption bands for BCSO suggest that this oil was the richer source of the phenolic compounds and anthocyanins, with an absorption maxima at around 460–560 nm (Figure 1c,d). Interestingly, total polyphenols in seed oil extracted from dried raspberry pomaces ranged between 178.1 and 204.7 mg of gallic acid equivalents/kg [21].

Specifically, absorbance bands at approximately 470 nm and 670 nm (Figure 1c,d), characteristic of carotenoids and chlorophylls, respectively, were observed in the UV-VIS spectra of both investigated oils. Nevertheless, chlorophyll pigments, such as the pheophytin responsible for the greenish color and carotenoid components like lutein and carotene homologs from yellow pigments dominated in BCSO. However, the presence of an absorption band at about 446 nm indicates a somewhat higher amount of α-carotene in RSO. Compared with the results of other authors [18,20], the oils recovered from black currant seeds and raspberry seeds had similar concentrations of total carotenoids (13.2–38.0 and 23 mg/100 g, respectively). Moreover, Oomah et al. [18] reported a negligible content of green pigments, mainly chlorophyll, in the 600–750 nm range (absorbance = 0.003 ± 0.007) in raspberry seed oil. Other researchers have recently recorded similar UV-VIS spectra containing qualitative and quantitative data on raspberry seed oils and black currant seed oil [18,29,30].

On the other hand, spectrophotometric measurements of the UV absorption at 232 nm (K_232_), 268 nm (K_268_), and 315 nm (K_315_) of 0.1% *n*-hexane solutions of RSO and BCSO were quantified indicators of conjugated dienes, trienes, and tetraenes, respectively, formed from hydroperoxides of polyunsaturated fatty acids and their oxidation products.

It is noteworthy that the specific coefficient value K_232_ (40.50), mainly indicating the conjugated dienes and carbonyl compounds, was significantly higher in RSO than the K_232_ (39.65) value for BCSO (Table 1, Duncan test).

Similarly, the indices K_268_ (9.00 and 8.24), which measure conjugated trienes and secondary products of oxidation (carbonyl compounds) that absorb at λ = 268 nm, differed significantly for both studied oils. In addition, RSO amounted to significantly higher conjugated tetraenes than BCSO (K_315_ = 4.86 and 1.65, respectively). The determined K_232_, K_268_, and K_315_ indices suggest that BCSO revealed a significantly better oxidative state than RSO. Notably, the absorption maximum did not always appear at 315 nm, or even at another constant wavelength, or was translocated—usually to a longer wavelength, up to 320 nm. These features are not unexpected since absorption in this region of the spectrum is a result of the absorbances of numerous conjugated tetraenes formed by auto-oxidative sequences as well as several other components, such as tocopherols, sterols, pigments, and their oxidation products. Moreover, the spectrum shape in this region is strongly affected by the high absorption intensity at 232 and 268 nm.

In comparison with the results of Ohma et al. [18], the conjugated diene value of raspberry seed oil was 0.837 due to the high linoleic acid content. In contrast, conjugated triene was not detected, indicating the absence or very low levels of linolenate oxidation in oil.

### 2.2. Color Measurement Results

Packaging color influences product perception; therefore, the color of the prepared films was tested. The color parameters (L*, a*, and b*) quantified using the CIELab color space analysis and total color difference (ΔE) of the prepared alginate films with and without RSO and BCSO are presented in Table 2.

As can be seen, the lightness L* and a* parameter of sodium alginate film were highest, while the b* parameter was lowest, close to 0, which means that this film was transparent and colorless. The incorporation of oil marginally reduced lightness, suggesting that the film transparency was maintained. Parameter b* increased considerably from −0.14 for alginate film to 2.93–5.80 for doped films, indicating a significant contribution of yellow in the film color, whereas a* decreased slightly from −1.92 for alginate film to −2.20–−2.65 for films with oils, indicating a green contribution to the film color. Films with RSO had slightly higher a* and b* parameters, indicating these films’ more intensive yellow color than those with BCSO (Table 2, Duncan test). UV-VIS spectra of RSO showed a somewhat higher content of carotenoids and a significantly lower chlorophyll content in this oil than in BCSO. Thus, films with RSO had a more yellow hue than films with BCSO, which revealed a more green tint. Moreover, the amount of carotenoids in BCSO was slightly lower than the content of chlorophylls, which influenced the color of films loaded with this oil. Generally, films enriched with higher oil content had worse transparency, lower a* and higher b* values, and more intense color. However, the opposite effect was noticed for films with BCSO.

In addition, ΔE values ranged between 3.10 and 6.08 for all studied films, indicating a noticeable color difference compared to alginate film without oils (Table 2).

Mutlu [31] studied gelatin/sodium alginate films with grape seed oil nanoemulsion. The studies showed that all films were transparent (L* ~ 94) and had a green-yellow hue (a* ~ −0.90, b* ~ 6). Adding grape seed oil to the gelatin/alginate film only slightly influenced the film color. In another study, films made of carboxymethylcellulose and alginate-formed films with soybean oil were prepared [32]. The L* value was about 70 for native film, and it negligibly decreased for films with soybean oil. Parameters a* and b* increased significantly, indicating the yellow shade of the films with soybean oil. Nehchiri et al. [33] obtained alginate films with sunflower oil and linseed oil. The neat alginate film was described by L* ~ 93, a* = 1.16, b* = 3.40, whereas the films with oils were characterized by L* ~ 92, a* = −1.24–−1.36 and b* = 3.58–4.16, indicating a yellowish-greenish tint similarly to our results. Moreover, similar results were presented by Khorrami et al. [34], who studied alginate films with nanostructured lipid carriers (NLC) and observed the yellow color of films with increasing concentrations of NLC.

### 2.3. Scanning Electron Microscopy Results

**Figure 2:**
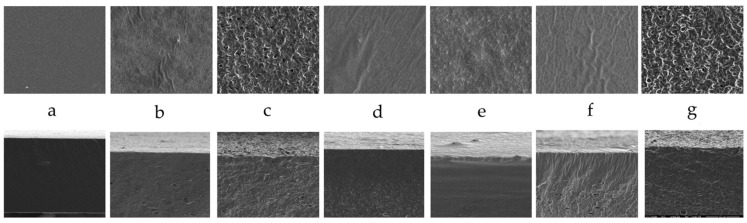
SEM images of the surfaces (**top**) and fractures (**bottom**) of the sodium alginate films with the studied oils; Viewed at a magnification of 2500×: (**a**) Alg+G; (**b**) Alg+G+RSO (25%); (**c**) Alg+G+RSO (50%); (**d**) Alg+G+BCSO (25%); (**e**) Alg+G+BCSO (50%); (**f**) Alg+G+(RSO+BCSO) (25%); (**g**) Alg+G+(RSO+BCSO) (50%).

Microphotographs of the prepared films’ surfaces (top row) and cross-sections (bottom row) are depicted in Figure 2. A smooth and plane surface was a characteristic of the alginate film. Adding RSO and/or BCSO to the biopolymeric film resulted in entirely different film structures, depending on the amount and type of oil. Long, narrow hills and round plane valleys were seen on the film’s surface with RSO (25%), but no holes or cracks were detected. The film surface with a higher oil content became more developed, with many holes, bubbles, protrusions, and tortuosities. The cross-sections of these films were similar and presented relatively evenly distributed small flattened cavities in the whole bulk of the films, indicating an even distribution of RSO. In the film with higher oil content, the distances between holes were smaller, and the structure was more folded due to the higher amount of oil.

BCSO altered the alginate surface differently. The film’s surface with a lower oil content revealed long, slim parallel streaks and many disordered convex arrangements. In contrast, the film with a higher content of this oil revealed a surface composed of deformed round depressions separated by bulges. The cross-section of the film with 25% BCSO revealed a compact area below the surface, and the bottom part of the film abounded in different-sized holes. The film’s cross-section with a higher BCSO content showed a more uniform structure; however, only in the middle part were horizontal cavities detected.

In a previous publication [27], we studied the properties of these films and found that the thickness of the films with BCSO was thinner than those with RSO, which resulted from the partly compact structure of the films with BCSO, as seen in SEM images.

Khorrami et al. [34] observed the porous structures of cross-sections of alginate films with lipid nanoparticles. Nehchiri et al. [33] obtained alginate films with sunflower oil and linseed oil by dividing them into oil and then *n*-hexane. The films showed smooth surfaces with some cracks due to the shrinkage process during drying; the interior of all films was plane and compact.

### 2.4. Thermal Analysis Results

**Figure 3:**
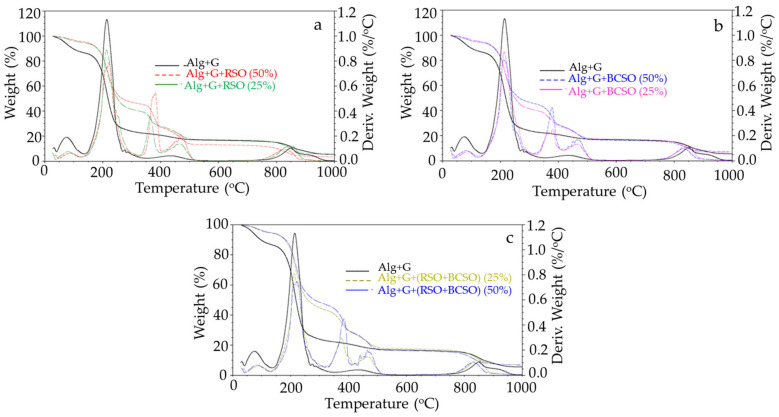
Thermogravimetric thermograms and the first derivative of mass change of control sodium alginate film with glycerol (Alg+G) before and after 25% and 50% additions of (**a**) raspberry seed oil (RSO), (**b**) black currant seed oil (BCSO), and (**c**) mixtures of raspberry seed oil and black currant seed oil (RSO+BCSO).

Thermogravimetric studies were conducted to check the thermal stability of the alginate films incorporated with the two different oils from fruit seeds. The TG and DTG curves are presented in Figure 3, while the various thermal parameters obtained from thermogravimetric (TG) and the first derivative of mass (DTG) thermograms of sodium alginate film with and without 25% and 50% of RSO, BCSO, and mixtures of the two are listed in Table 3.

We observed that the plasticized sodium alginate film decomposed in four stages. In the first stage, at temperatures up to about 130 °C, the sample lost approximately 13% of its mass related to water release. The second and main stage, with 64.5% mass loss in the range of 130–300 °C, resulted from the biopolymer decomposition with the formation of carbonized material and sodium carbonate [35]. In this stage, glycerol was also removed [36,37]. The third stage, with about 5% mass loss in the temperature range of 350–550 °C, was due to the decomposition of charred material [35]. The last step, which took place in temperatures between 750 and 1000 °C and in which the sample lost 10.8% of its mass, was caused by the decomposition of sodium carbonate and the formation of sodium oxide and carbon dioxide [38]. The residue of 5.6% at 980 °C was probably sodium oxide [35,38].

The TG and DTG curves of alginate films with oils showed five, quite well-separated stages of decomposition, and all these films showed a similar course (Figure 3). The first stage of the films’ decomposition to 125 °C was related to the release of water, which constituted about 5% in all the samples, less than in alginate film. There were similar results for carboxymethylcellulose/alginate films with soybean oil [32].

The second stage, in the temperature range of 125–300 °C, was assigned to the glycerol evaporation and alginate decomposition and the formation of charred material and sodium carbonate. Moreover, films with lower oil content lost about 54% of their mass. In comparison, films with higher oil content lost about 49% of their mass, less than those without oils, suggesting less efficient alginate decomposition in the presence of oil. Thus, the efficiency of the decomposition process of alginate was affected by the presence of oils in the films.

Next, the third stage, in which the films lost 12–15% and 18–19% of mass for lower oil content and higher oil content, respectively, could be associated with the decomposition of surfactant in the temperature range of 310–410 °C [34,39,40].

The fourth stage, in the temperature range of 410–510 °C, was related to the decomposition of oil and carbonaceous material and the formation of sodium carbonate. The samples with lower oil content lost about 9–10% of their mass; samples with higher oil content lost about 9–14%. Khorrami et al. [34] observed a peak in temperature range from 400 to 560 °C in the alginate films with nanostructured lipid carriers, which was associated with the lipids’ decomposition and evaporation.

The residue, i.e., sodium oxide, after the decomposition of sodium alginate, was 3.8–7.4% at 980 °C for the studied samples. In another study, residue at 600 °C was higher for carboxymethylcellulose/alginate films with soybean oil compared with neat film, indicating the higher thermal stability of the films with soybean oil [32].

## 3. Materials and Methods

### 3.1. Chemicals and Materials

Sodium alginate (Alg) was acquired from Büchi Labortechnik AG (Flawil, Switzerland). Glycerol (G) was bought from Avantor Performance Materials Poland S.A. (Gliwice, Poland), surfactant Tween 80 was supplied by Greenaction (Kielce, Poland), and *n*-hexane was provided by Chempur (Piekary Slaskie, Poland). All other chemicals and solvents, purchased from Merck Sp. z o. o. (Warszawa, Poland), were of analytical grade and used without further purification.

However, raspberry seed oil (*Rubus idaeus*) (RSO) and black currant seed oil (*Ribes nigrum*) oil (BCSO) were obtained from Etja S.C. (Elbląg, Poland).

### 3.2. Characterization of Raspberry Seed Oil and Black Currant Seed Oil

The UV-VIS spectra of RSO and BCSO in *n*-hexane were recorded with a spectrophotometer UV-2600i (Shimadzu, Kyoto, Japan) in the 200–800 nm range. The oil spectra were used for the qualitative analysis of the oils. These spectra were recorded for different oil dilutions in *n*−hexane, and the most representative spectra were chosen for presentation.

Conjugated diene, triene, and tetraenes values in oils added to alginate films as active ingredients were measured using the same spectrophotometer as the absorbance of a 1% solution of each oil in *n*-hexane (no additional dilutions were made) at 232, 268, and 315 nm, respectively, in a 1 cm quartz cell using *n*-hexane as a blank according to the ISO 3656:2011 method [41]. The absorbance value was divided by the weight of the oil sample (Abs/Weight_oil_).

### 3.3. Preparation of Active Alginate Films

The films for the study were prepared by pouring the solution on levelled polystyrene Petri dishes. First, a sodium alginate of 2% (*w*/*v*) aqueous solution was prepared, to which was added 2.5% (*w*/*v*) of glycerol. Independently, mixtures of oils from fruit seeds with surfactant (Tween 80) were prepared in a 1:1 ratio. Next, an appropriate amount of oil mixture with a surfactant was introduced to the 30 cm^3^ alginate solution and mixed on a magnetic stirrer. Then, the whole beaker content was cast into the Petri dishes, which were 9 cm in diameter. The solvent evaporated, and the films were ready to be removed from the dishes. The film’s weight ratio of biopolymer to oil was 2:1 (50%) or 4:1 (25%).

### 3.4. Optical, Morphological, and Thermal Properties of Alginate Films

#### 3.4.1. Surface Color Measurement

The color of the tested film samples was assessed with a Colorimeter CL 400 (Courage & Khazaka, Köln, Germany). The CIE LAB trichromatic color model L*, a*, and b* parameters were determined for each film in three replications. In this model, L* is defined as lightness, a* means the chromaticity parameter from red to green, and b* represents the chromaticity parameter from yellow to blue. The total color difference (ΔE) was calculated using the following formula:

$$\Delta E=\sqrt{{\left(L-L*\right)}^{2}+{\left(a-a*\right)}^{2}+{\left(b-b*\right)}^{2}}$$

where: L*, a*, b* are parameters related to the reference, which was plasticized sodium alginate film. The measurements were conducted on a white sheet of paper.

#### 3.4.2. Scanning Electron Microscopy

The surface and cross-section pictures of the obtained films were acquired with a scanning electron microscope (SEM), model 1430 VP, 2001 (LEO Electron Microscopy Ltd., Cambridge, UK). The acceleration voltage was 5 kV or 10 kV. Before measurements, the studied alginate films were covered with an approximately 10 nm layer of gold and palladium.

#### 3.4.3. Thermal Analysis

The thermal stability of the obtained films was tested on a thermoanalyzer SDT 2960 Simultaneous DSC/TGA analyzer (TA Instruments, New Castle, DE, USA) in a nitrogen atmosphere with a heating rate of 10 °C/min to 1000 °C. Thermogravimetric (TG) curves and derivative of thermogravimetric (DTG) curves were used for the determination of the characteristic parameters, such as T_0_ (°C), the temperature of the beginning of the process, T_f_ (°C), the temperature of the end of the process, T_max_, the temperature at which the process runs at a maximal rate, Δm (%), the mass loss during the process, and a residue (%) at 980 °C.

### 3.5. Statistical Analysis

Analyses were carried out in three or nine replicates (three times for three samples of the same film) and were presented as mean ± standard deviation (SD). The mean comparisons to analyze the significant differences were computed using the Duncan test at a 95% confidence interval using analysis of variance (ANOVA) with Statistica 8.0 software (StatSoft, Tulsa, OK, USA).

## 4. Conclusions

For the first time, bioactive-rich oils with low amounts of oxidative products extracted from fruit residues such as raspberry and blackcurrant seeds were incorporated into an alginate sodium matrix to fabricate active films. The addition of raspberry seed oil, black currant seed oil, and mixtures of the two in different concentrations enhanced the alginate films’ functional properties. The incorporation of oils to alginate films marginally reduced their lightness, although films enriched in higher oil content had worse transparency. Moreover, films with various amounts of raspberry seed oil were more yellow in color than those with black currant seed oil due to a somewhat higher content of carotenoids and the significantly lower chlorophyll content in this oil in comparison with black currant seed oil. Furthermore, scanning electron microscopy images confirmed that the presence of raspberry seed oil, black currant seed oil, and their mixtures in biopolymeric matrices resulted in entirely different film morphologies and structures depending on the amount and type of oil. The film surface with a higher raspberry seed oil content became more developed, with many holes, bubbles, protrusions, and tortuosities, whereas the film with a higher amount of black currant oil revealed a surface composed of deformed round depressions separated by bulges. In contrast, incorporating oils from fruit residues marginally affected the beginning of the thermal degradation of sodium alginate. However, these functional components reduced the efficiency of the thermal decomposition of alginate in the first stage.

This study demonstrated that incorporating raspberry seed oil and black currant seed oil into alginate films improved their optical, morphological, and thermal properties. Our previous report highlighted the fact that both oils enhanced the mechanical and antioxidant properties of the enriched alginate films. In summary, the developed active alginate films with fruit seed oils incorporated demonstrate potential as eco-friendly food packaging materials. Moreover, adding oils extracted from fruit wastes to the production of biodegradable active packaging materials reduces fruit solid residues and leads to their higher valorization.

## Figures and Tables

**Table 1:** The spectroscopic indices K_232_, K_268_, and K_315_ quantitatively characterize conjugated dienes, trienes, and tetraenes in the investigated oils.

Oil Sample	K_232_ ± SD	K_268_ ± SD	K_315_ ± SD
Raspberry seed oil	40.50 ± 0.09 ^b^	9.00 ± 0.19 ^b^	4.86 ± 0.20 ^b^
Black currant seed oil	39.65 ± 0.08 ^a^	8.24 ± 0.21 ^a^	1.65 ± 0.17 ^a^

* *n* = 3; SD—standard deviation; different letters within the same column (a,b) indicate significant differences between concentrations of conjugated polyenes in two oils (Duncan test, *p* < 0.05).

**Table 2:** Color parameters (L*, a*, b*) of the prepared alginate films.

Sample	L* ± SD	a* ± SD	b* ± SD	ΔE
Alg+G	95.48 ± 0.23 ^b^	−1.92 ± 0.15 ^a^	−0.14 ± 0.19 ^a^	
Alg+G+RSO (25%)	94.61 ± 0.34 ^a^	−2.20 ± 0.21 ^b^	4.62 ± 0.28 ^c,d^	4.85
Alg+G+RSO (50%)	94.21 ± 0.42 ^a^	−2.31 ± 0.17 ^b^	4.83 ± 0.56 ^d^	5.14
Alg+G+BCSO (25%)	94.60 ± 0.34 ^a^	−2.65 ± 0.31 ^d^	4.62 ± 0.77 ^c,d^	4.89
Alg+G+BCSO (50%)	94.54 ± 0.54 ^a^	−2.40 ± 0.16 ^b,c^	4.22 ± 0.75 ^c^	4.49
Alg+G+(RSO+BCSO) (25%)	95.08 ± 0.79 ^b^	−2.20 ± 0.30 ^b^	2.93 ± 0.54 ^b^	3.10
Alg+G+(RSO+BCSO) (50%)	94.36 ± 0.36 ^a^	−2.58 ± 0.21 ^c,d^	5.80 ± 0.30 ^e^	6.08

* *n* = 9; SD—standard deviation; different letters within the same column (a–e) indicate significant differences between color parameters of the studied films (Duncan test, *p* < 0.05).

**Table 3:** The thermal parameters obtained from DTG curved (T_max_) and TG curves (mass loss, residue at 980 °C) for plasticized alginate films with raspberry seed oil (RSO), black currant seed oil (BCSO), their mixtures, and for alginate film without oils.

Sample	T_max1_, °C/Δm_1_, %	T_max2_, °C/Δm_2_, %	T_max3_, °C/Δm_3_, %	T_max4_, °C/Δm_4_, %	T_max5_, °C/Δm_5_, %	Residue at 980 °C, %
Alg+G	74/13.6	214/64.5	433/5.5		850/10.8	5.6
Alg+G+RSO (25%)	80/5.1	213/54.7	364/12.5	464/10.8	848/11.1	5.8
Alg+G+RSO (50%)	87/4.2	211/49.8	376/19.0	466/13.9	828/9.3	3.8
Alg+G+BCSO (25%)	87/5.1	213/54.5	380/14.2	463/9.1	846/10.6	6.5
Alg+G+BCSO (50%)	83/5.2	210/49.2	377/18.7	469/10.6	833/8.9	7.4
Alg+G+(RSO+BCSO) (25%)	87/5.0	213/51.4	376/15.7	466/10.0	849/10.5	7.0
Alg+G+(RSO+BCSO) (50%)	84/5.3	212/47.6	382/18.7	464/12.1	831/9.4	6.9

## Data Availability

The data presented in this study are available in the article.
